# BAC and RNA sequencing reveal the brown planthopper resistance gene *BPH15* in a recombination cold spot that mediates a unique defense mechanism

**DOI:** 10.1186/1471-2164-15-674

**Published:** 2014-08-11

**Authors:** Wentang Lv, Ba Du, Xinxin Shangguan, Yan Zhao, Yufang Pan, Lili Zhu, Yuqing He, Guangcun He

**Affiliations:** State Key Laboratory of Hybrid Rice, College of Life Sciences, Wuhan University, Wuhan, China; National Key Laboratory of Crop Genetic Improvement, Huazhong Agricultural University, Wuhan, China

**Keywords:** Brown planthopper, *BPH15*, Recombination coldspot, Candidate gene, RNA sequencing, Transcriptome, Defense mechanism

## Abstract

**Background:**

Brown planthopper (BPH, *Nilaparvata lugens* Stål), is the most destructive phloem-feeding insect pest of rice (*Oryza sativa*). The BPH-resistance gene *BPH15* has been proved to be effective in controlling the pest and widely applied in rice breeding programs. Nevertheless, molecular mechanism of the resistance remain unclear. In this study, we narrowed down the position of *BPH15* on chromosome 4 and investigated the transcriptome of *BPH15* rice after BPH attacked.

**Results:**

We analyzed 13,000 BC_2_F_2_ plants of cross between susceptible rice TN1 and the recombinant inbred line RI93 that carrying the *BPH15* gene from original resistant donor B5. *BPH15* was mapped to a 0.0269 cM region on chromosome 4, which is 210-kb in the reference genome of Nipponbare. Sequencing bacterial artificial chromosome (BAC) clones that span the *BPH15* region revealed that the physical size of *BPH15* region in resistant rice B5 is 580-kb, much bigger than the corresponding region in the reference genome of Nipponbare. There were 87 predicted genes in the *BPH15* region in resistant rice. The expression profiles of predicted genes were analyzed. Four jacalin-related lectin proteins genes and one LRR protein gene were found constitutively expressed in resistant parent and considered the candidate genes of *BPH15*. The transcriptomes of resistant *BPH15* introgression line and the susceptible recipient line were analyzed using high-throughput RNA sequencing. In total, 2,914 differentially expressed genes (DEGs) were identified. BPH-responsive transcript profiles were distinct between resistant and susceptible plants and between the early stage (6 h after infestation, HAI) and late stage (48 HAI). The key defense mechanism was related to jasmonate signaling, ethylene signaling, receptor kinase, MAPK cascades, Ca^2+^ signaling, PR genes, transcription factors, and protein posttranslational modifications.

**Conclusions:**

Our work combined BAC and RNA sequencing to identify candidate genes of *BPH15* and revealed the resistance mechanism that it mediated. These results increase our understanding of plant–insect interactions and can be used to protect against this destructive agricultural pest.

**Electronic supplementary material:**

The online version of this article (doi:10.1186/1471-2164-15-674) contains supplementary material, which is available to authorized users.

## Background

The brown planthopper (BPH; *Nilaparvata lugens* Stål) is a typical phloem-feeding insect and a major pest of rice (*Oryza sativa*). At this time, 24 BPH-resistance genes have been identified in rice, 20 of which are located on chromosomes [1]. Resistance genes *BPH14* and *BPH15* were introgressed from wild rice *Oryza officinalis* [2]. These two genes showed significant resistance to BPH and have been broadly employed in rice breeding programs [3, 4]. Recently, *BPH14* was isolated using a map-based cloning strategy, and it was found to encode a coiled-coil, nucleotide-binding, and leucine-rich repeat (CC-NB-LRR) protein that activates the SA signaling pathway [5]. In rice breeding, *BPH15* shows a greater resistance effect than *BPH14* and *BPH18* when introgressed into the elite indica rice 9311 and hybrid rice [6]. *BPH15* is located on the short arm of chromosome 4, where 4 BPH-resistance genes are clustered [7, 8].

Plant responses to insect attack are correlated to the mode of feeding [9, 10]. Gene expression profiles suggested that defense mechanisms against BPH differ from those against chewing insects. Genes involved in macromolecule degradation and plant defenses were found to be upregulated, whereas those involved in photosynthesis and cell growth were downregulated after BPH infestation [11]. Quantitative proteomics results revealed that proteins involved in JA synthesis, oxidative stress response, *β*-glucanases, and kinases showed significant changes in expression in response to BPH feeding [12]. Nitric oxide was used by plants as a signaling molecule and plays a role in the rice tolerance response to BPH feeding [13]. Callose deposition on the sieve plates occluded the sieve tubes and inhibited continuous feeding by BPH in resistant lines; thus, the death of BPH on resistant lines was the result of starvation and not poisoning [14]. Nevertheless, complete transcriptional analysis of the BPH response genes remains unavailable, and more comprehensive differential expression profiles are required to better understand the molecular mechanism of BPH resistance in rice.

To clone *BPH15* and increase our understanding of the molecular mechanism of resistance, we backcrossed the resistant plant carrying the *BPH15* locus to susceptible rice and developed the backcrossed populations. *BPH15* was located in a recombination cold spot of 580-kb. High-throughput RNA sequencing represents the latest and most suitable tool for characterizing the transcriptome [15]. We then applied deep RNA sequencing to investigate the transcriptomes of *BPH15* introgression line and susceptible recipient line. In the sequenced *BPH15* region, four jacalin-related lectin (JRL) domain proteins and a LRR family protein were considered candidate *BPH15* genes. The molecular mechanism of resistance to BPH was also explored by comparing differentially expressed genes (DEGs) between resistant and susceptible rice.

## Results

### BPH resistance gene *BPH15*is located in a recombination cold-spot region

**Figure 1:**
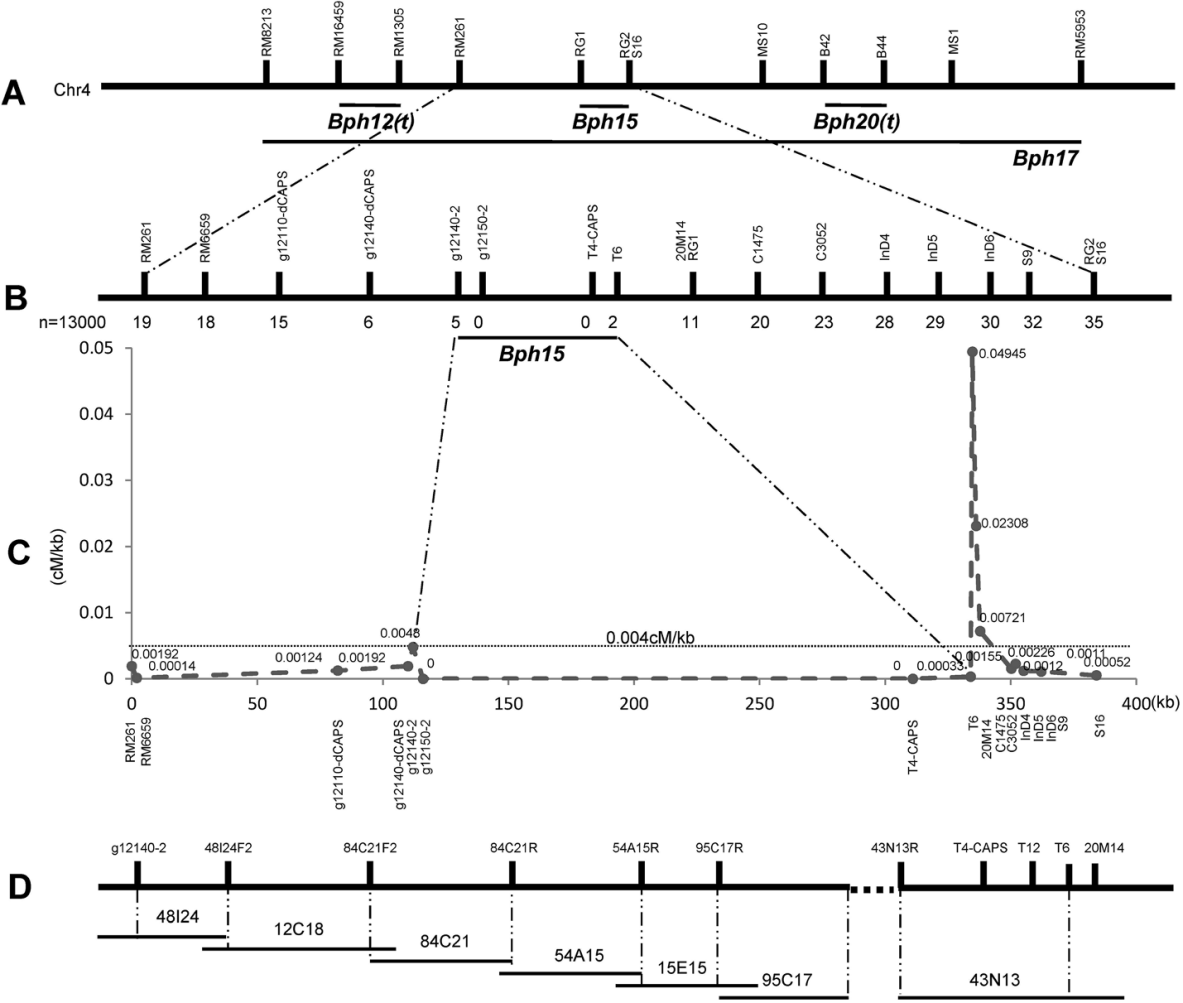
**Fine mapping of the** ***BPH15*** **locus. A**, The marker positions of four previously mapped BPH resistant genes. **B**, Screened recombinant information in the BC_2_F_2_ family. Numbers under the linkage map indicate the number of recombinants detected between the marker and *BPH15. BPH15* was mapped to the region between markers g12140-2 and T6. **C**, Recombination frequencies between each adjacent DNA marker. The dashed line represents genome average 0.004 cM/kb value. **D**, Physical map assembled by PCR-screened BAC clones. The dashed line represents the gap where no BAC clones overlapped.

We previously mapped the major BPH-resistance gene on the short arm of chromosome 4 between molecular markers RG1 and RG2 using the F_2_ population of cross between RI93, a recombinant inbred line that carrying the *BPH15* gene from original resistant donor B5, and susceptible rice TN1 (Figure 1A) [7]. To fine-map the gene, the resistant rice line YHY15 carrying the *BPH15* locus was selected from the F_2_ population and backcrossed to TN1 to develop mapping populations (Additional file 1). After genotyping BC_1_F_2_ and phenotyping the BC_1_F_3_ populations, *BPH15* was mapped between markers RM261 and S16 (Figure 1A). We further screened 13,000 BC_2_F_2_ plants for recombination between RM261 and S16, and 54 recombination events were identified. The average genome-wide recombination rate (the ratio of total genetic map length in centimorgans divided by the genome size in base pairs) is about 0.004 cM/kb in rice [16]. Chromosome intervals between RM261 and S16 showed a much lower recombination rate (0.0005 cM/kb). After genotyping the recombinant plants using newly developed markers (Additional file 2) and phenotyping the fixed recombinant plants, *BPH15* was mapped to a 0.0269 cM interval defined by g12140-2 and T6 (7 recombinants in 13,000 plants) (Figure 1B; Additional file 3-1). A high recombination rate was observed just outside of the *BPH15* region. Specifically, the 0.7-kb fragment from marker T6 to 20M14 is a hot spot with a genetic recombination rate of 0.049 cM/kb, much higher than the whole genome level (Figure 1C). Afterward, we identified 61 recombinants from 10,000 BC_4_F_2_ plants, similar to BC_2_F_2_ plants, and no crossover was found in the *BPH15* region (Additional file 3-2). Based on these results, *BPH15* is located in a recombination cold-spot region, and identifying candidate *BPH15* genes using conventional analysis of recombination is difficult.

### High level of sequence diversity in the *BPH15*region

**Figure 2:**
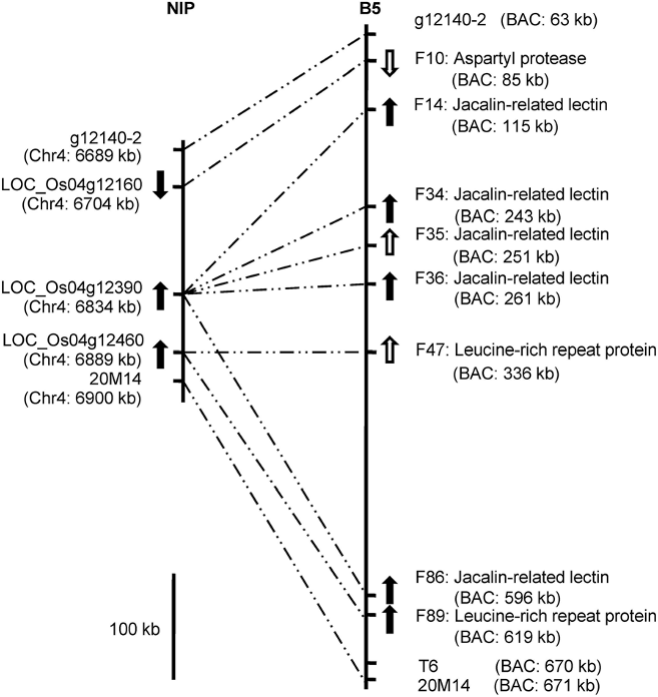
**The** ***BPH15*** **region relationship between Nipponbare and BPH-resistant rice B5.** The solid and hollow arrows represent expressed and unexpressed genes and their direction, respectively. Markers g12140-2, T6 and 20M14 relate to the region in Figure 1D. The notes in brackets represent gene location on the chromosome or assembled 700-kb BAC sequences. LOC_Os04g12160: aspartic proteinase nepenthesin-2 precursor; LOC_Os04g12390: transposon protein, containing a jacalin lectin domain; LOC_Os04g12460: leucine rich repeat family protein. The genes location are listed in Additional file 4.

The physical distance between the markers g12140-2 and T6 is approximately 210-kb in the Nipponbare genome. To detect the actual length and identify the genes in the region spanning the *BPH15* locus, we constructed a genomic BAC library of long insertion fragments for the resistant rice B5, the original donor of *BPH15*. The markers g12140-2 and T6 were used to screen BAC clones by amplification of the BAC DNA pools for an initial chromosome walking. Using five steps of walking, the physical map of the *BPH15* region was assembled, and the shortest path consisted of seven BAC clones (Figure 1D). A gap existed between the BAC 95C17 and BAC 43N13. The seven BAC clones were sequenced and included a 700-kb region. Finally, we mapped the *BPH15* locus between g12140-2 and newly developed marker T12 (Additional file 3-1; Figure 1D), and the physical distance was at least 580-kb according to sequenced BAC clones. Eighty-seven genes within this 580-kb region were annotated using online FGENESH software (from F8 to F94) and 70 genes were TE-related genes (Additional file 4-1). There were 31 annotated genes in the 210-kb Nipponbare sequence according to MSU 7.0 and 21 genes were TE-related genes (Additional file 4-2). Excluding several functional genes (Figure 2), the sequences in this region of the two rice genotypes are highly diverse. Based on these results, the sequence of this region in the resistant rice genome differed significantly from the corresponding region in the reference genome of Nipponbare; i.e., the 210-kb fragment in Nipponbare was replaced by a much larger fragment containing several repeat sequences in resistant rice. Thus, developing codominant molecular markers for this region was difficult. The high level of sequence diversity in the *BPH15* region explained the heavy suppression of recombination in this region.

### BPH-responsive transcript profiles are distinct in resistant *BPH15*introgression line and susceptible recipient line

To identify the *BPH15* candidate genes and understand the molecular mechanism of resistance, the expression profiles of *BPH15* introgression line (R) and susceptible recipient line (S) were determined using deep RNA sequencing. RNAs extracted from rice samples at the early stage (6 h after infestation, HAI), late stage (48 HAI) and control (0 HAI) of two rice lines were sequenced. As a result, 198 million paired-end sequence reads of 100 bp in length were generated in six samples. After removing low-quality reads, a total of 150 million high-quality clean reads remained, of which 90.48–92.15% were aligned to the reference genome using TopHat (Table 1).

**Table 1:** **Statistics of sequencing reads and alignment to the reference genome**

Samples	Raw data reads	Raw data base (bp)	High-quality reads	High-quality base (bp)	Percentage of alignment (%)
S0	37,061,382	3,706,138,200	28,008,690	2,357,352,788	91.84
S6	24,715,554	2,471,555,400	18,742,423	1,581,929,269	91.38
S48	32,339,240	3,233,924,000	24,661,359	2,081,307,813	90.48
R0	39,251,668	3,925,166,800	29,661,746	2,489,101,005	92.15
R6	35,214,634	3,521,463,400	26,576,329	2,234,567,794	91.39
R48	29,490,134	2,949,013,400	22,533,122	1,903,312,211	91.84
all	198,072,612	19,807,261,200	150,183,669	12,647,570,880	-

Percentage of alignment = high quality reads aligned to genome/high-quality reads.

**Figure 3:**
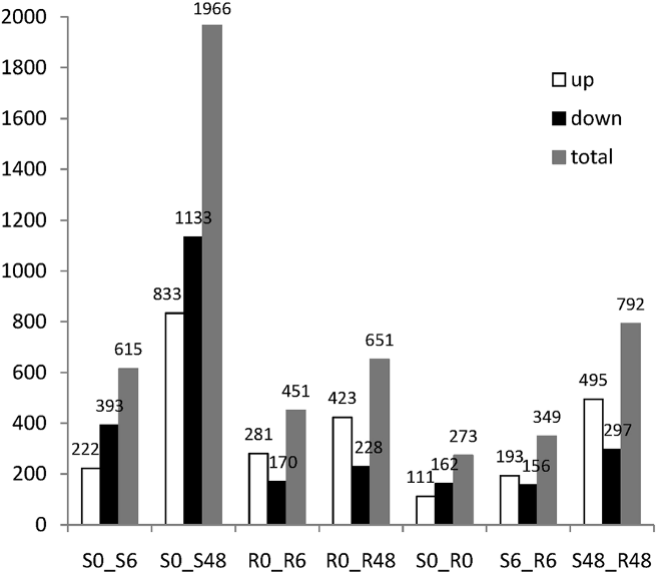
**Contrast between upregulated and downregulated DEGs in all comparisons.**

One fundamental use of transcriptome sequencing is analysis of differentially expressed genes (DEGs) between samples [15]. In our study, we defined DEGs as the transcripts showing at least a 1.5-fold change of the FPKM (fragments per kilobase of exon per million fragments mapped) (log_2_FC ≥ 0.585 or log_2_FC ≤ –0.585) and *P*-value < 0.05. In total, 2,914 DEGs were detected among seven comparisons: S0_S6, S0_S48, R0_R6, R0_R48, S0_R0, S6_R6, and S48_R48 (Additional file 5). In susceptible rice, 615 and 1966 DEGs were found in S0_S6 and S0_S48 comparisons, respectively. In contrast, in resistant rice, the DEGs numbers were 451 and 651 in R0_R6 and R0_R48, respectively (Figure 3).

**Figure 4:**
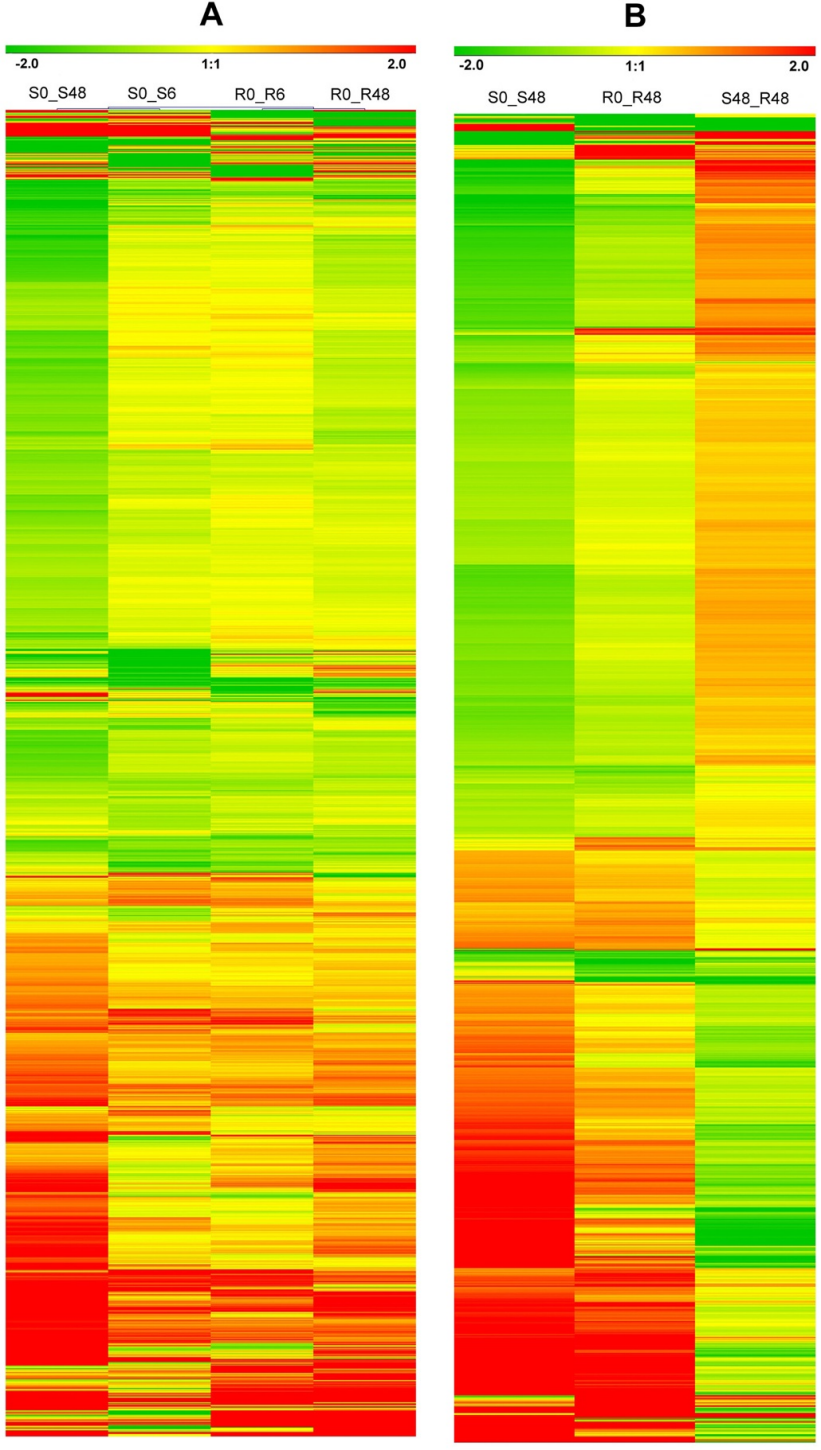
**Hierarchical clustering analysis of DEGs based on the log ratio of FPKM data.** The color key represents FPKM normalized log_2_ transformed counts. Red indicates upregulated DEGs and green denotes downregulated DEGs. Each column shows a comparison and each row represents a gene. **A**, DEGs of S and R. **B**, DEGs at 48 HAI.

DEGs in susceptible and resistant rice at 6 HAI and 48 HAI were hierarchically clustered, and the heat map is shown in Figure 4A. The majority of DEGs had similar expression patterns among four comparisons, showing consistent upregulation or downregulation, although not all *P*-values for the four comparisons were below 0.05. To compare the two rice genotypes, DEGs exclusively at 48 HAI were selected and hierarchically clustered. Most of these DEGs had lower amplitude of variation in the resistant genotype compared to the susceptible genotype (Figure 4B). The k-means clustering analysis results also supported the conclusion (Additional file 6).

High-throughput technologies such as microarray and sequencing methods generate enormous amounts of data, but individual functional annotation of all DEGs remains challenging. Pathway-based analysis to characterize the interaction between genes increases our understanding of the biological function of DEGs [17]. DEGs assigned to MapMan pathways and important classifications are listed in Additional file 7 and provided in Table 2. To verify the RNA-Seq results, the expression of 23 DEGs was analyzed by quantitative polymerase chain reaction (qPCR) with gene-specific primers (Additional file 2). Additional file 8 provided detailed RNA-seq fold-change values for every DEG and their qPCR results of three biological replicates. The qPCR results were consistent with RNA-seq data, since the genes displayed similar fold-changes with a correlation ratio of *R*^2^ = 0.971 (Additional file 9).

**Table 2:** **Pathway classification by MapMan**

Pathways	S-all		R-all		S_R-all	
	U	D	U	D	U	D
Ethylene	21	7	14	3	4	8
Jasmonate	4	2	2	0	0	0
Salicylic acid	4	0	2	0	0	0
Receptor kinase	20	5	9	3	5	4
Ca^2+^ signaling	14	8	6	3	1	3
Biotic stress	35	8	13	2	4	20
Wounding	4	1	4	0	1	1
AP2/EREBP TF	15	3	9	0	1	8
bHLH TF	6	1	7	0	0	1
Zinc finger family TF	12	1	5	1	4	4
WRKY domain TF	10	1	7	0	2	1
Protein synthesis, targeting and folding	6	148	3	30	71	9
Protein degradation-ubiquitin	26	4	17	1	5	9
Protein posttranslational modification	25	12	12	0	3	8
Bowman–Birk-type bran trypsin inhibitor	7	1	6	1	0	5
Glucan endo-1,3-*β*-glucosidase	11	2	6	0	1	9
Photosynthesis	8	97	4	27	38	5
Tetrapyrrole synthesis	0	23	0	10	14	1
Major CHO synthesis	2	6	2	2	2	0
Major CHO degradation	6	3	3	1	3	2
Lipid metabolism	5	51	6	5	26	5
TCA, mitochondrial electron transport	2	22	3	1	1	3

S-all: total DEG number in comparisons of S0_S6 and S0_S48; R-all: DEG number in comparisons of R0_R6 and R0_R48; S_R-all: DEG number in comparisons of S0_R0, S6_R6 and S48_R48.U: upregulated; D: downregulated.

Hormone signaling pathways play pivotal roles in plant defense [18, 19]. In this study, the majority of ethylene synthesis genes and ethylene signal transduction genes, such as ACO (LOC_Os02g53180) and ERF (LOC_Os02g43790), were upregulated in two rice genotypes, but the number of DEGs in resistant rice was less than that in susceptible rice (Additional file 7; Table 3). This result suggested that BPH feeding activated the ET signaling pathway in susceptible rice. Jasmonate synthesis genes, such as lipoxygenase LOX (LOC_Os08g39850), allene oxidase synthase AOS2 (LOC_Os03g12500), and 12-oxophytodienoate reductase OPR1 (LOC_Os06g11210) were upregulated in susceptible rice, which suggests that BPH attack induces the JA signaling pathway (Additional file 7; Table 3). Four SA carboxyl methyltransferase (SAMT) genes were upregulated in susceptible rice (Additional file 7), which can reduce SA content by forming MeSA from SA in the plants [20]. Other SA synthesis and signaling genes were not identified in DEGs. We measured the SA content in leaf sheath surrounding the stem from plants exposed to BPH for 0, 3, 6, 24, and 48 h using gas chromatography–mass spectrometry (GC-MS; Additional file 10). No significant difference in SA levels was observed between the BPH-infested and control plants or between resistant and susceptible plants, except that SA levels in 48 HAI susceptible rice were significantly lower than in resistant rice. The lower SA content may be caused by upregulated SAMT genes expression. These results indicated that the SA signaling pathway may not be activated in *BPH15*-mediated resistance or during the basal defense of susceptible rice.

**Table 3:** **Representative pathway genes**

Pathways	Representative genes	Transcript	S0_S6	S0_S48	R0_R6	R0_R48
Ethylene	ACO	LOC_Os02g53180.1	-	U	-	-
	ERF	LOC_Os02g43790.1	-	U	-	-
Jasmonate	LOX	LOC_Os08g39850.1	-	U	-	-
	AOS2	LOC_Os03g12500.1	-	U	-	-
	OPR1	LOC_Os06g11210.1	-	U	-	-
Salicylic acid	SAMT	LOC_Os11g15040.1	U	U	-	U
Receptor kinase	Receptor-like protein kinase 5	LOC_Os02g13510.1	-	U	-	U
	BAK1	LOC_Os03g32580.1	-	U	-	-
	XA21	LOC_Os11g36180.1	-	-	-	U
MAP kinase	MAPK	LOC_Os03g17700.1	-	U	-	-
Ca^2+^ signaling	OsCML15	LOC_Os05g31620.1	-	U	-	-
	Calmodulin-binding protein	LOC_Os12g36910.1	-	U	-	U
Biotic stress	PR1a	LOC_Os07g03710.1	-	U	-	-
	PR1b	LOC_Os01g28450.1	-	U	-	U
	PR3	LOC_Os03g30470.1	-	U	-	-
	PR4b	LOC_Os11g37960.1	-	U	-	-
	PR9	LOC_Os07g48020.1	-	U	-	U
	PR10	LOC_Os12g36880.1	-	U	U	U
Wounding	WI12	LOC_Os03g18770.1	U	-	-	-
TF	EREBP	LOC_Os03g08500.1	-	U	-	-
	Basic helix–loop–helix protein	LOC_Os09g31300.1	-	U	-	U
	C2H2 zinc finger protein	LOC_Os05g37190.1	-	U	-	U
	C3H zinc finger protein	LOC_Os07g38090.1	-	-	-	-
	WRKY domain TF	LOC_Os01g14440.1	-	U	-	-
Pr. synthesis	tRNA synthetase	LOC_Os01g54020.2	-	D	-	-
	40S ribosomal protein	LOC_Os03g18570.1	-	D	-	-
	60S ribosomal protein	LOC_Os05g06310.1	-	D	-	-
	Translation initiation factor	LOC_Os05g49970.2	-	D	-	-
Pr. targeting	Mitochondrial import translocase	LOC_Os02g48610.2	-	D	-	-
Pr. folding	Chaperonin	LOC_Os06g09679.1	-	D	-	D
Pr. degradation	Ubiquitin family protein	LOC_Os02g06640.1	-	U	-	-
	OsFBL7	LOC_Os02g10700.1	U	U	-	-
Pr. modification	Protein phosphatase	LOC_Os02g13100.1	-	U	-	-
	Calcium-dependent protein kinases	LOC_Os07g05620.2	-	U	-	U
	CRK5	LOC_Os04g56430.1	-	U	-	-
	CRK6	LOC_Os03g16960.1	-	U	-	-
	Serine/threonine protein kinase	LOC_Os02g01730.1	-	U	-	-
Trypsin inhibitor	BBTI5	LOC_Os01g03360.1	U	U	U	-
Glucosidase	GNS1	LOC_Os05g31140.1	-	U	-	-
	GNS4	LOC_Os01g71670.1	-	U	-	U
	GNS5	LOC_Os01g71340.1	-	U	-	U
PS light reaction	PS I reaction center subunit	LOC_Os03g56670.1	-	D	-	-
	PS II reaction center protein	LOC_Os01g71190.1	D	D	-	D
	ATP synthase	LOC_Os07g32880.1	-	D	-	-
PS Calvin cycle	Ribulose bisphosphate carboxylase	LOC_Os12g17600.1	-	D	-	-
Tetrapyrrole	Aminolevulinic acid dehydratase	LOC_Os06g49110.1	-	D	-	-
CHO synthesis	Starch synthase	LOC_Os04g53310.1	D	-	D	-
CHO degradation	*β*-amylase	LOC_Os03g22790.1	U	U	U	-
Lipid synthesis	Acetyl-CoA carboxylase	LOC_Os05g22940.1	-	D	-	-
	Glycerol-3-phosphate acyltransferase	LOC_Os01g63580.1	-	D	-	-
TCA	Citrate synthase	LOC_Os01g19450.1	-	D	-	-

In total, 35 and 13 proteins responding to biotic stress were upregulated in susceptible and resistant rice, respectively, such as SCP-like extracellular protein PR1a (LOC_Os07g03710), PR1b (LOC_Os01g28450), chitinase family protein PR3 (LOC_Os03g30470), wound-induced protein PR4b (LOC_Os11g37960), PR9 (LOC_Os07g48020), and pathogenesis-related *Betv1* family protein PR10a (LOC_Os12g36880) (Tables 2 and 3). However, the number of DEGs in resistant rice was lower than in susceptible rice (Additional file 7). Herbivore-induced callose deposition on the sieve plates of rice is an important mechanism for host resistance. *β*-1,3 glucan hydrolase genes are activated and cause unplugging of the sieve tube occlusions in susceptible plants [14]. The majority of this gene family were upregulated in susceptible and resistant rice, such as GNS1 (LOC_Os05g31140), GNS4 (LOC_Os01g71670), and GNS5 (LOC_Os01g71340), but the number of DEGs was lower in resistant rice (Additional file 7; Tables 2 and 3). In total, 148 of 154 protein synthesis, protein targeting, and protein folding related genes were downregulated in susceptible rice, but only 30 were downregulated in resistant rice (Tables 2 and 3). Thus, the reprogramming of protein synthesis and secretion machinery was significantly affected in the susceptible lines but not in the resistant lines, which was suggestive of significant damage in susceptible rice.

**Figure 5:**
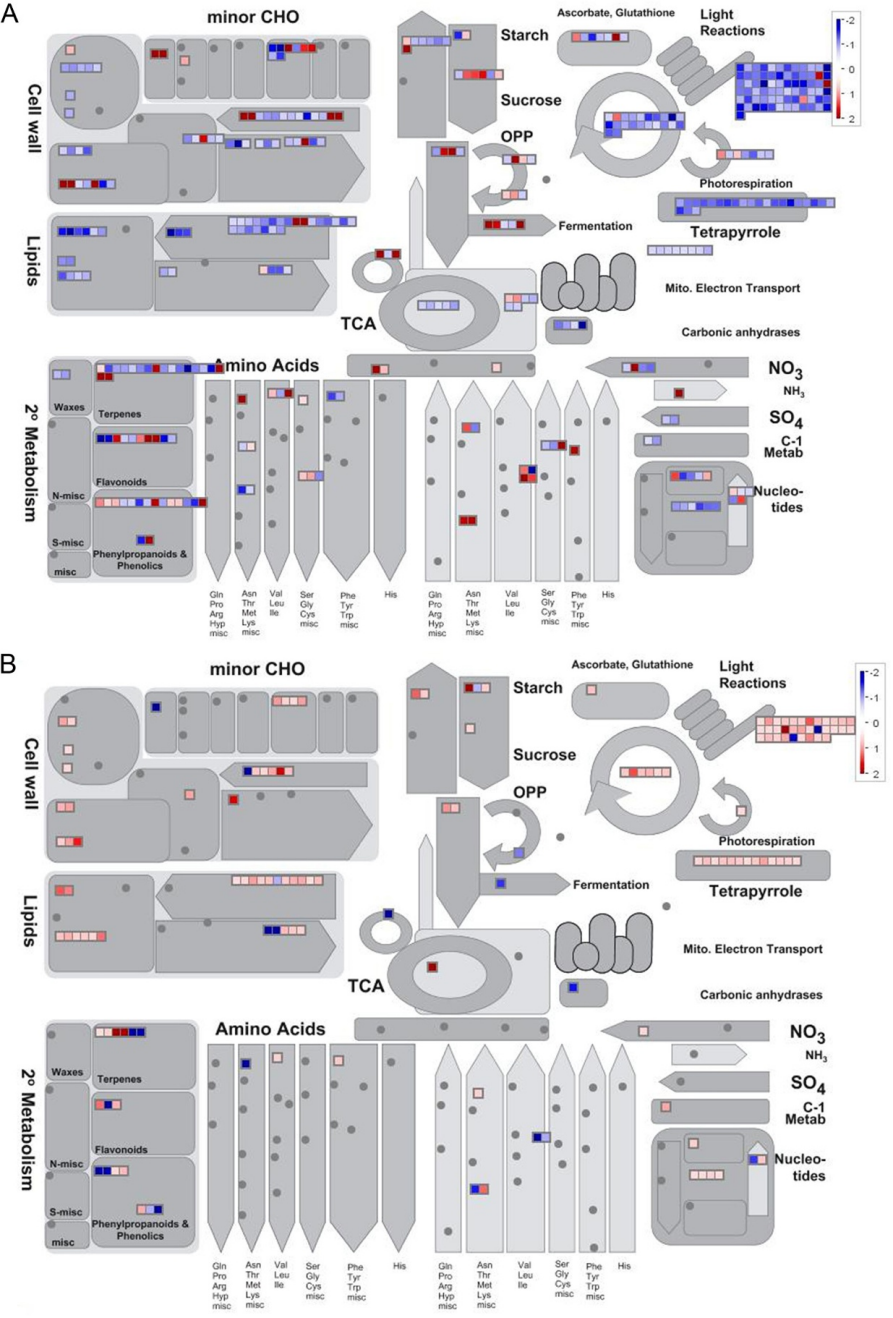
**MapMan overview of metabolism.** Individual genes are represented by small squares. The color key represents FPKM normalized log_2_ transformed counts. Red indicates upregulation and dark blue denotes downregulation. **A**: S0_S48 DEG; **B**: S48_R48 DEG.

DEGs assigned to photosynthesis (PS), tricarboxylic acid cycle (TCA), mitochondrial electron transport/ATP synthesis, major carbohydrate (CHO) metabolism and lipid metabolism were highly downregulated in susceptible rice. However, these genes had low amplitude of variation in resistant rice (Additional file 7; Table 2). So, these genes were upregulated in resistant rice as compared between with those in suseptible rice (Figure 5). The PS comprised genes coding for proteins involved in the photosynthetic electron transport chain, photorespiration, Calvin cycle, and Photosystem I and II complexes. We observed a general downregulation of genes associated with photosynthetic processes at 6 HAI and 48 HAI in both susceptible and resistant rice, but with a higher number of DEGs in susceptible genotype at 48 HAI. BPH response included genes involved in the primary metabolism, mainly related to carbohydrate and lipid metabolism. These genes were predominantly downregulated in susceptible rice after BPH infestation, but the carbohydrate degradation genes were upregulated (Table 3). Our data indicated that BPH attack generally represses photosynthesis-related genes in susceptible rice leaves, as well as those involved in primary metabolism.

### BPH-responsive transcript profiles are distinct in early and late feeding stages of two rice genotypes

As shown in Figure 3, the number of DEGs at 6 HAI was less than 48 HAI in both the resistant and susceptible rice. This demonstrated that both rice genotypes experienced weak damage in the early stages of BPH feeding, and resistant rice showed a relatively normal physiological status compared to susceptible rice. In addition, the amplitude of variation at 6 HAI was less than that at 48 HAI in both rice genotypes (Figure 4A; Additional file 6).

To understand key biological processes involved in the rice response to BPH, we used singular enrichment analysis of agriGO to identify enriched GO terms. All compared DEGs showed significant GO terms, excluding S0_R0 and S6_R6 (Additional file 11). As a result, 40 significant GO terms were found in total DEGs in susceptible line (union of S0_S6 and S0_S48), 25 in resistant line (union of R0_R6 and R0_R48), and 35 in comparison of the two lines (union of S0_R0, S6_R6, and S48_R48) (Additional files 12, 13 and 14). Investigation of the rice transcriptome following BPH feeding revealed the activation of a wide and complex response, and the transcriptional reconfiguration involved a broad range of biological processes. The 40 GO terms were mainly distributed in several categories, including photosynthesis, primary metabolism, secondary metabolism, response to stimulus, cell wall, and ribosome. GO terms of important biological functions were compared for significance in early and late feeding stages of two rice genotypes (Table 4). Common GO terms significant in all comparisons and unions were response to stimulus, response to abiotic stimulus, response to stress, cell wall, plastid, and thylakoid. The 48 HAI-specific GO terms were response to biotic stimulus, photosynthesis, generation of precursor metabolites and energy, ribosome, and intracellular organelle. Several GO terms, including macromolecule biosynthetic process, translation, gene expression, secondary metabolic process, and biosynthetic process, were significant only in 48 HAI-susceptible rice and the corresponding S48_R48. All GO terms were significant in 48 HAI-susceptible rice, and showed smaller *P*-values. Overall, during BPH infestation, the most rapid gene expression adjustments were observed in the categories of response to stimulus and cell wall; the DEGs involved in photosynthesis, response to biotic stimulus, and ribosome gradually increased and became significant at late feeding stage.

**Table 4:** **Significant GO terms**

GO accession	GO terms	R0_R6	S0_S6	R0_R48	R-all	S0_S48	S-all	S48_R48	S_R-all
GO:0050896	Response to stimulus	7.90E-07	3.50E-08	1.00E-08	1.80E-11	2.10E-14	5.50E-17	2.10E-06	2.40E-09
GO:0009628	Response to abiotic stimulus	1.10E-06	3.10E-08	7.90E-08	4.10E-10	1.10E-20	6.00E-24	1.10E-12	3.40E-16
GO:0006950	Response to stress	7.90E-06	1.10E-07	7.10E-08	1.60E-10	1.40E-12	5.80E-16	6.30E-07	7.70E-10
GO:0005618	Cell wall	2.60E-06	1.10E-06	1.50E-03	8.60E-08	2.90E-12	3.70E-15	3.10E-08	6.60E-09
GO:0009536	Plastid	3.80E-06	1.60E-07	3.40E-10	8.40E-12	4.20E-83	3.90E-79	1.80E-31	1.20E-28
GO:0009579	Thylakoid	1.90E-05	2.50E-12	4.30E-20	1.70E-18	1.00E-102	1.60E-97	8.70E-52	1.10E-47
GO:0015979	Photosynthesis	-	-	8.90E-09	2.20E-07	4.90E-42	8.90E-41	5.00E-21	1.30E-17
GO:0009607	Response to biotic stimulus	-	-	4.70E-07	5.20E-08	2.40E-13	4.80E-13	5.70E-08	4.70E-08
GO:0006091	Generation of precursor metabolites and energy	-	-	4.80E-04	8.40E-04	4.50E-21	2.30E-20	4.00E-11	3.30E-11
GO:0005840	Ribosome	-	-	2.00E-06	2.70E-04	2.60E-24	9.50E-23	4.70E-16	4.20E-16
GO:0043229	Intracellular organelle	-	-	6.10E-04	6.30E-04	3.90E-23	4.20E-21	2.30E-09	6.10E-09
GO:0009059	Macromolecule biosynthetic process	-	-	-	-	3.50E-15	9.40E-13	6.00E-13	1.40E-12
GO:0006412	Translation	-	-	-	-	3.50E-15	9.40E-13	6.00E-13	1.40E-12
GO:0010467	Gene expression	-	-	-	-	3.00E-10	2.10E-08	6.00E-10	1.10E-09
GO:0019748	Secondary metabolic process	-	-	-	-	2.30E-04	1.40E-05	4.60E-04	1.50E-06
GO:0009058	Biosynthetic process	-	-	-	-	2.30E-04	4.20E-04	1.20E-03	3.30E-04

R-all: union of R0_R6 and R0_R48; S-all: union of S0_S6 and S0_S48; S_R-all: union of S0_R0, S6_R6, and S48_R48.

### Common defense-related genes in two rice genotypes

Receptor kinase, kinase cascades, and Ca^2+^ signaling-related genes are important components of signal transduction and play roles in transmitting resistance signals to downstream response genes [21]. Twenty and nine receptor kinases in susceptible and resistant rice were upregulated, respectively (Table 2). This included LRR family protein receptor-like protein kinase 5 (LOC_Os02g13510), brassinosteroid insensitive 1-associated receptor kinase 1 precursor BAK1 (LOC_Os03g32580), and receptor kinase XA21 (LOC_Os11g36180) (Table 3). These upregulated receptor kinase genes suggested that signal perception was activated after BPH feeding. The intracellular concentration of Ca^2+^, an important second messenger, typically increases in response to biotic or abiotic stress. In our studies, the majority of Ca^2+^ signaling-related genes were upregulated, such as calmodulin-related calcium sensor protein OsCML15 (LOC_Os05g31620) and calmodulin-binding protein (LOC_Os12g36910) (Table 3), which suggests that the rice defense response to BPH involved Ca^2+^ influx. Two MAP kinase genes LOC_Os03g17700 and LOC_Os06g48590 were upregulated (Additional file 7). Protein posttranslational modification genes were also upregulated, such as cysteine-rich receptor-like protein kinase CRK5 (LOC_Os04g56430), CRK6 (LOC_Os03g16960), calcium/calmodulin dependent protein kinases (LOC_Os07g05620), serine/threonine protein kinase (LOC_Os02g01730), and protein phosphatase (LOC_Os02g13100) (Table 3). CRK5 and CRK6 were detected based on a proteomic approach to evaluate *BPH15* [12]. These upregulated genes suggest that a resistance signal is transmitted to downstream regulatory networks after rice perceives BPH.

Transcription factors (TFs) play an important role in the defense response [22]. We detected approximately 200 TF DEGs in two rice genotypes, and more were found in susceptible rice than resistant rice (Tables 2 and 3; Additional file 7). EREBP binds to the GCC box, a conserved ethylene responsive promoter element found in many defense-related genes [23]. A systematic expression analysis of rice WRKY revealed a large number of WRKY DNA-binding proteins involved in the transcriptional activation of defense-related genes in response to rice pathogens [24]. Overexpression of the *OsWRKY89* gene enhanced resistance to the rice blast fungus and white-backed planthopper [25].

Protein degradation-related genes were upregulated in both rice genotypes (Table 2). The protein degradation system is thought to be responsible for selective degradation of proteins folded incorrectly as a result of stress [26]. In response to abiotic stress, wound-responsive proteins WI12 (LOC_Os03g18770) were upregulated. Wound-response pathways were also detected in BPH-feeding rice [27]. Eight Bowman–Birk-type bran trypsin inhibitor precursor genes play a role in resistance to BPH in *BPH14*-containing rice [5], and we also found that they were upregulated in this research (Additional file 7; Table 3).

### Candidate genes of *BPH15*

We searched for expressed genes within the 87 genes predicted in the 580-kb *BPH15* region using RNA sequencing data of resistant rice. We identified five jacalin-related lectins (F14, 34, 35, 36, and 86) and two LRR family proteins (F47 and 89) (Additional file 4-1; Figure 2), most of which showed higher FPKM values than other predicted genes. These genes had similar FPKM values and their expression levels were not significantly different at three time points (Additional file 4-1). These genes were examined using reverse transcription-PCR (RT-PCR), and we found that four jacalin-related lectins (F14, 34, 36, and 86) and a LRR family protein (F89) were constitutively expressed in resistant rice before and after BPH feeding (Figure 2). Other functional genes (one aspartyl protease family protein and six receptor-like protein kinases), which were only detected in a few raw fragments (Additional file 4-1), were not detected in RT-PCR. The majority of transposon protein and retrotransposon protein predicted genes were also detected in a few raw fragments. We then detected all potentially expressed genes (FGENESH and RiceGAAS predicted genes) and found that no other genes were expressed. Note that the four jacalin-related lectin genes showed similar BLASTp results in the Nipponbare genome, and the corresponding gene LOC_Os04g12390 also contained a jacalin domain. However, the gene was annotated as an En/Spm subclass transposon protein in the MSU Rice Genome Annotation Project Release 7.0 (Additional file 4-1). We inferred that these orthologous genes originated from the same ancestral gene (Figure 2). The LRR protein F89 expressed in resistant rice corresponded to LOC_Os04g12460, which also predicted as a LRR gene. Based on these results, the candidate gene *BPH15* represents one or a few of these expressed genes, which is being verified based on complementation tests.

## Discussion

The wild relatives of crops contain numerous genes of economic importance that are critical for genetic improvement of crops and understanding the mechanism of traits under control by these genes [28]. The use of genes from wild relatives to improve crop performance is well established [29]. Genetic improvement of crop plants by developing introgressed lines originating in wild relatives usually results in a reduction in recombination rates within introgressed segments [30]. By using molecular markers, a lot of introgression of chromosome segments from *O. officinalis* to *O. sativa* had been observed [31–33], though the mechanism of introgression is poorly understood. Important traits such as insect and disease resistance have been transferred into cultivated rice [32, 34]. BPH resistance gene *BPH15* was introgressed from wild rice *O. officinalis* [35]. In this study, *BPH15* was located in a recombination cold spot, which is similar to the powdery mildew resistance gene *Mla* in barley [36].

The C genome of *O. officinalis* is estimated to be 651 Mb, which is larger than the 430 Mb of the A genome of cultivated rice *O. sativa*. This 580-kb region of *BPH15*, which contains numerous repeat sequences, in the resistant rice genome corresponds to the 210-kb fragment in the Nipponbare genome. Excluding several functional genes (Figure 2), the sequences in this region of the two genotypes are highly diverse. More TE-related genes appear in the *BPH15* region of resistant rice than in the corresponding region of Nipponbare (Additional file 4-1). The important roles of retrotransposons to modify genome size, remodel genome structure, and displace gene functions in the plant genome have been observed in several studies, which indicates that retrotransposons are an important driving force in genome evolution [37]. Previous studies demonstrated that retroelement insertions contributed to the C genome expansion of *O. officinalis* [38] [Oryza Map Alignment Project (OMAP), http://www.omap.org], which might cause divergence in the *BPH15* region between *O. officinalis* and *O. sativa*. The diversity in the intergenic, repetitive DNA regions should be responsible for the low chromosome pairing and recombination between *O. sativa* and *O. officinalis* [33]. In this experiment, introgression of the highly diverse 580-kb segment is the primary cause of the low recombinant rate, making it difficult to isolate the *BPH15* gene using positional cloning. These difficulties were also encountered in other processes of gene cloning. The recombination repression of nematode resistance gene *Mi* was also thought to be a consequence of the alien origin of the DNA segment [39]. Another reason for suppressed recombination in the *BPH15* region is that the locus is located near the centromere. A study found that chromosomal recombination at the centromere core and surrounding regions on six chromosomes was completely suppressed [16], and a substantial reduction in recombination was observed in the regions of the short arm and the pericentromeric region of chromosome 4 [40].

We also observed a recombination hot spot located just outside the *BPH15* segment, where the local recombination rate is much higher than the whole genome average value in the BC_2_F_2_ family. Recombination hot spots in many species show significant relationships with gene density, GC content, and specific gene functional categories [41]. The 0.7-kb recombination hot-spot between markers T6 and 20M14, located just on the right side of the 580-kb replacement fragment, shows average base composition of the overall chromosome (data not shown). Based on these observations, the recombination hot-spot region showed no correlation with its base properties but position (close to the large replacement fragment). This is important for future studies on the position relationship between recombination hot spots and cold spots to analyze the mechanism of activation and inactivation of recombination.

Identifying candidate genes in an uncharacterized genomic region with no recombination is difficult. In our study, BAC clone sequencing of the *BPH15* region and deep RNA sequencing of resistant rice were combined to analyze candidate resistance genes. Eighty-seven genes annotated in this 580-kb region exist between marker g12140-2 and T12, and most of them are TE-related genes. Moreover, only four jacalin-related lectins and a LRR domain protein were expressed in the resistant rice. Many plant lectins have anti-insect potential, some have strong insecticidal properties. Transgenic rice plants expressing lectins showed resistance to BPH and other insects [42]. Hessian fly-responsive gene 1 (*Hfr-1*) is a novel jacalin-like lectin gene from wheat (*Triticum aestivum*) plants that responds to infestation by Hessian fly (*Mayetiola destructor*) larvae [43]. Lectins, are also known to play important roles in defense responses against pathogens. A jacalin-related lectin-like gene (*TaJRLL1*) in wheat is a component of the plant defense system [44]. The mannose-binding lectin gene *CaMBL1* from pepper plays a key role in the regulation of plant cell death and defense responses through the induction of downstream defense-related genes and SA accumulation after the recognition of microbial pathogens [45]. Some lectin receptor-like kinases have been implicated in rice resistance to pathogens and herbivores [46, 47]. As the four jacalin-related lectin genes in the *BPH15* region expressed in the resistant rice, we speculated that one or multiple of these lectin genes are *BPH15* candidates and function in resistance to BPH. The jacalin-related lectin genes clustered in *BPH15* region might evolve from ancient duplications driven by TE elements, as what has been shown in other resistance gene evolution in plant [48]. Another candidate gene is the LRR domain protein in the *BPH15* region. The majority of resistance (R) genes that have been cloned belong to the NB-LRR family and the first cloned BPH-resistance gene *BPH14* is a NB-LRR member [5, 49, 50]. Therefore, the five candidate genes are currently being verified using complementation tests.

RNA sequencing of the *BPH15* introgression line and the susceptible recipient line provided transcriptome data on the mechanism of resistance conferred by *BPH15*. In our study, several rice genes have been associated with the BPH response for the first time, which provides information on signal transduction pathways and defense responses elicited by the BPH in rice. We analyzed plants at 6 HAI and 48 HAI, before the development of visible symptoms, and compared the expression profiles between early and late stages of infestation. The BPH extracts large volumes of phloem sap to attain adequate sugar, which should influence expression of genes involved in carbon assimilation and mobilization. The transcriptional downregulation of photosynthetic and primary metabolism related genes appears to be a universal adaptive response of plants to phloem-feeding insects and represents a shift in resource allocation from growth to basal defense [19]. In susceptible rice, BPH can significantly reduce photosynthetic rates in host plants, but resistant plants show few symptoms of damage and grow normally after 2 days of feeding [11]. In this experiment, photosynthesis, TCA, CHO metabolism, lipid metabolism, and protein synthesis related genes were downregulated in susceptible rice. However, resistant rice containing *BPH15* shows lower expression changes, suggesting that the resistant rice has a stronger tolerance than susceptible rice. The early stage and late stage showed significantly different expression profiles. The majority of DEGs show a less significant change and fewer DEGs are observed at 6 HAI, which may be due to minor damage during the short BPH feeding time. Significant GO terms of photosynthesis, generation of precursor metabolites and energy, ribosome, and intracellular organelle only appeared during the late stages. Previous comparative analyses of expression profiles of proteins in *BPH15* rice leaf sheaths in response to infestation by the BPH found that in response to stress caused by pest invasion, plants develop a basal defense, which appeared stronger in the susceptible lines compared with resistant lines [12]. In this report, the amplitude of variation in resistance rice was lower than that in susceptible rice (Figure 4), in which the upregulated genes in susceptible rice may function as a basal defense. The majority of upregulated ethylene signals, receptor kinases, biotic response PR genes, and transcription factor genes in susceptible rice showed larger fold-changes than in resistant rice (Additional file 7; Table 2). *BPH14* and *Mi-1* both activate an SA-dependent resistance pathway after BPH and nematode feeding [5, 51]. However, the SA pathway may not be present in the *BPH15* resistance mechanism according to our sequencing data and SA content measured using GC-MS. The key defense mechanism found in our study was related to jasmonate signaling, ethylene signaling, receptor kinase, MAPK cascades, Ca^2+^ signaling, PR genes, TFs, and protein posttranslational modifications. Receptor kinase XA21 (LOC_Os11g36180) was upregulated only in resistant rice (Additional file 7). These exclusively upregulated DEGs in resistant rice increase our understanding of the molecular mechanism of resistance in *BPH15*. In addition, the candidate genes (JRL and LRR genes) may participate in the unique defense mechanism of *BPH15*. These lectin proteins may have insecticidal properties or deterrent activity to the BPH. The JRL and LRR genes may also perceive BPH feeding as a receptor to transduce defense signals to downstream genes. However, this hypothesis requires further confirmation and candidate gene complementation tests.

## Conclusions

The BPH-resistance gene *BPH15* was mapped to a recombination cold-spot region. The high level of sequence diversity in the *BPH15* region explained the heavy suppression of recombination in this region. We found that BPH-responsive transcript profiles were distinct between resistant and susceptible plants and between the early stage and late stage. Susceptible rice showed more DEGs and larger amplitude of variation than resistant rice after BPH feeding. More genes were regulated at the late stage than those at the early stage. The key defense mechanism in resistant *BPH15* and susceptible recipient rice was related to jasmonate signaling, ethylene signaling, receptor kinase, MAPK cascades, Ca^2+^ signaling, PR genes, transcription factors, and protein posttranslational modifications. Four jacalin-related lectin proteins genes and one LRR protein gene predicted in the *BPH15* region were expressed constitutively and considered candidate *BPH15* genes. These results increase our understanding of plant–insect interactions and can be used to protect against this destructive agricultural pest.

## Methods

### Fine mapping of *BPH15*

YHY15, a resistant rice line containing the *BPH15* locus from RI93/TN1 F_2_ population [7], was backcrossed to the susceptible rice TN1 to develop populations to fine-map the gene (Additional file 1). The BC_1_F_2_ plants were used for genotype analysis and BC_1_F_3_ lines harvested from each of the BC_1_F_2_ plants were assayed for BPH resistance. To fine-map *BPH15*, 13,000 BC_2_F_2_ plants were screened to obtain recombinants between PCR markers RM261 and S16. The selected recombinant plants were self-pollinated and used to select fixed recombinants (BC_2_F_3_), which showed homozygous resistant and susceptible genotypes on both sides of the crossover. The seeds of fixed recombinants (BC_2_F_4_) were used to phenotype using the seedling bulk test method. Twenty seeds of each line were sown in a 20-cm-long row with 2.5 cm between rows in a plastic box. YHY15 and TN1 were randomly sown among the BC_2_F_4_ plants as controls. At the third-leaf stage, the seedlings were infested with BPH nymphs at a level of eight insects per seedling. When all of the TN1 seedlings had died (scored as 9), each seedling was given a score of 0, 1, 3, 5, 7, or 9 according to Huang et al. [35]. The resistance score of each BC_2_F_3_ plant was then inferred from the scores of the seedlings in the corresponding BC_2_F_4_ plants. At the same time, additional molecular markers (CAPS, dCAPS, InDel, and SSR) were developed in the region to genotype the recombinants plants. Briefly, primer pairs were designed for single-copy regions in Nippobare and used to amplify YHY15 and TN1. Products were cloned into the T-vector for sequencing, and the polymorphisms between YHY15 and TN1 were used to develop molecular markers. *BPH15* was located in a smaller region according to the genotype and phenotype of recombinants plants. To identify more recombinant lines, BC_4_F_2_ was also screened in the region.

### BAC sequencing of the *BPH15*region

A genomic BAC library for resistant rice B5, the original donor of *BPH15*, was constructed. The library contained 36,864 clones with an average insert size of 130-kb. The coverage of the library was about 10 genome equivalents, which increases the probability of isolating unique rice genes or sequences in the library. BAC DNA pools were prepared and candidate clones were screened using PCR-based analysis [52]. Initial screening was performed using primers for the distal markers g12140-2 and T6. BAC ends were used to develop single-copy markers, rescreen the library, and extend the contig by chromosome walking. The low-copy sequences were efficiently identified using low-pass DNA sequencing of the BAC when the BAC end sequences were repetitive and could not be used for the walking step. Finally, overlapping BAC clones were assembled to the contig and sequenced by conventional BAC subclone library shotgun sequencing, assembling, and gap-filling. New markers were developed from BAC sequences to genotype the recombinant lines. The assembled sequence was annotated using the FGENESH (http://linux1.softberry.com/berry.phtml) and RiceGAAS (Rice Genome Automated Annotation System, http://ricegaas.dna.affrc.go.jp/) annotation systems.

### Preparation of RNA-Seq libraries

The BPH-resistant rice 9311(15), introgression lines containing *BPH15* [6] and susceptible recurrent parent 9311 were grown in pots (20 cm in diameter and 20 cm in height) with 30 plants per pot in a greenhouse, which was controlled to have 30 ± 2°C/14 h light (07:00–21:00) and 28 ± 2°C/10 h dark (21:00–07:00) cycles. At the three-leaf stage of rice (12 days after sowing), third-instar nymphs of BPH insects (biotype 1) were introduced to the rice plants at a density of eight insects per seedling. The stems were harvested after BPH infestation for 0 (uninfested control), 6, and 48 h. The six samples were named R0, R6, and R48 for the resistant genotype, and S0, S6, and S48 for the susceptible genotype in which the number represents the HAI. All time points begun at different times and stopped at the same time. The stems (5 cm in length) of 30 rice plants of each treatment were collected as a combined sample, quickly frozen in liquid nitrogen, and kept at –80°C until further analysis.

Total RNAs were prepared using RNAiso Plus according to the manufacturer’s protocol (TaKaRa Code: D9108A). All subsequent procedures, including mRNA purification, cDNA preparation, end repair of cDNA, adaptor ligation, and cDNA amplification were performed according to the manufacturer’s protocols accompanying the mRNA-Seq Sample Preparation Kit (Illumina). Each library had an insert size of 150 bp, and paired end sequences of 100 bp on each end (2*100 bp) were generated via Illumina HiSeq2000.

### Analysis of differentially expressed genes

The most recent rice genome and gene information (MSU Release 7.0) were downloaded from the Rice Genome Annotation Project (http://rice.plantbiology.msu.edu). The raw reads were cleaned by removing adaptor sequences, empty reads, low-quality sequences, and short reads. The remaining high-quality reads were aligned to the *Oryza sativa* genome by TopHat [53] (version: 2.0.6 http://tophat.cbcb.umd.edu). Cufflinks [54] (version: 2.0.2 http://cufflinks.cbcb.umd.edu) was used to calculate the FPKM value of every transcript. The *P*-value of different expression was calculated using Fisher’s exact test. We used *P* < 0.05 and the absolute value of log_2_FC ≥ 0.585 as the threshold to judge the significance of each gene expression difference. Cluster analysis and heat maps were performed with the Genesis software based on the hierarchical and k-means clustering method [55] (version: 1.7.6 http://genome.tugraz.at). GO analysis was performed using a Web-based tool agriGO [56] (http://bioinfo.cau.edu.cn/agriGO). For pathway analysis, we mapped all DEGs using the MapMan package [57] with the Osa_MSU_v7 mapping file and latest pathways downloaded from the official Web site (http://mapman.gabipd.org/web/guest/mapman).

### Real-time qPCR

Twenty-three genes of MapMan pathway classifications were selected for validation using real-time qPCR. Primer sets were designed with the Primer Premier 5 software. The qPCR was performed with the Sso Advanced SYBR Green Supermix and CFX96 Touch™ Real-Time PCR Detection System (Bio-Rad) following the manufacturer’s instructions. The results were analyzed using CFX Manager Software 2.1. EF-Tu (LOC_Os03g08020.1) was used as an internal control to standardize the results according to sequencing data. All results had three biological replicates and three technical replicates.

### Reverse transcription-PCR

The RNAs of *BPH15* introgression line and recipient line (0 and 48 HAI) were reverse transcribed to first strand cDNA. The expression of predicted genes in *BPH15* region was confirmed using RT-PCR. All primers were designed according to specific fragment of the genes. The PCR products of RT–PCR were verified by sequencing.

### Measurement of SA content using GC-MS

Four-week-old resistant and susceptible rice were infested with BPH nymphs for 0, 3, 6, 24, and 48 h. The leaf sheaths surrounding the stem were separated and frozen immediately in liquid nitrogen. The SA content was determined using a modified vapor-phase extraction method [58, 59]. Briefly, 100 mg of leaf sheath was ground to a fine powder in liquid nitrogen. After the addition of internal standards (^2^H_6_-SA, 300 ng), samples were extracted using a mixture of acetone and 50 mM citric acid (v/v = 7/3), and ethyl acetate. The supernatant was then dried using N_2_ and subsequently methylated with trimethylsilyldiazomethane. After stopping the methylation reaction with acetic acid in hexane, the samples were subjected to a vapor-phase extraction procedure using a volatile collector trap packed with Tenax absorbent and eluted with *n*-hexane. The eluted samples were then analyzed using GC-MS equipped with an AS3000 auto sampler (Trace GC Ultra/ISQ, Thermo Fisher Scientific). Compounds were separated on an Rtx-5MS (30 m × 0.25 mm × 0.25 *μ*m) column held at 50°C for 1 min after injection, after which the temperature was increased by 10°C min^–1^ to 180°C (4 min) and by 15°C min^–1^ to 280°C (5 min), with helium as the carrier gas (constant flow rate 1 mL min^–1^). Quantification of SA was completed by correlating the peak area (extracted ion) of the compound with that of the corresponding internal standard. Three independent biological replicates were sampled and all samples were measured three times with similar results.

### Availability of supporting data

The data sets supporting the results of this article are included within the article and its additional files. The RNA-Seq raw data were submitted to Short Read Archive at NCBI with accession number SRP039374.

## Electronic supplementary material

Additional file 1: **Pedigree flow chart.** (PDF 172 KB)

Additional file 2: **Primers used in this study.** (XLS 35 KB)

Additional file 3: **Recombinants genotype and phenotype.** (XLS 56 KB)

Additional file 4: **Cufflink analysis of sequenced BAC clones and annotation MSU release 7.0 of Nipponbare between markers g12140-2 and 20M14.** (XLS 66 KB)

Additional file 5: **Detailed information for all 2,914 DEGs.** (XLS 1 MB)

Additional file 6: **K-means clustering analysis of DEGs based on log ratio of FPKM data.** (PDF 401 KB)

Additional file 7: **The DEG assign to MapMan major pathways.** (XLS 289 KB)

Additional file 8: **Details on the qPCR data.** (XLS 40 KB)

Additional file 9: **RNA-seq concordance with real-time qPCR results.** (PDF 99 KB)

Additional file 10: **Measurement of salicylic acid content using gas chromatography–mass spectrometry.** (PDF 164 KB)

Additional file 11: **DEG enrichment of GO terms.** (XLS 46 KB)

Additional file 12: **Graphical result of significant GO terms of S-all.** (PDF 2 MB)

Additional file 13: **Graphical result of significant GO terms of R-all.** (PDF 472 KB)

Additional file 14: **Graphical result of significant GO terms of S_R-all.** (PDF 774 KB)
