# Supplementary material for: BAC and RNA sequencing reveal the brown planthopper resistance gene BPH15 in a recombination cold spot that mediates a unique defense mechanism
Source: BMC Genomics. 2014 Aug 11;15(1):674. doi: 10.1186/1471-2164-15-674 (PMC4148935; doi:10.1186/1471-2164-15-674)
Supplement: Supplementary file 1 — Additional file 1: Pedigree flow chart. (PDF 172 KB) [file 12864_2014_6374_MOESM1_ESM.pdf]

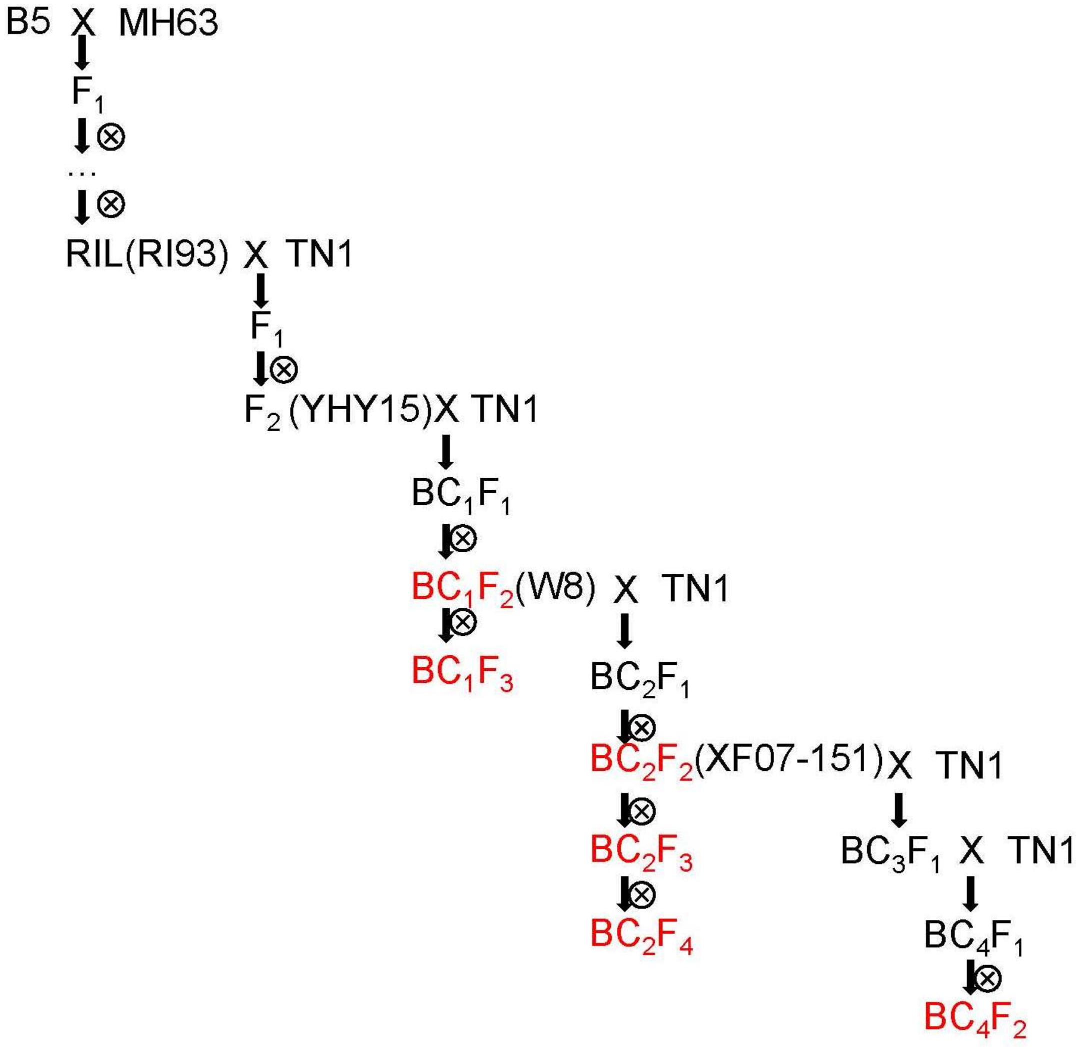

Additional file 1 Pedigree flow chart. RI93, a selected RIL (recombinant inbred line) of B5; YHY15, a F_2 plant carrying the *BPH15* locus was backcrossed to the susceptible rice TN1 to develop backcross mapping populations; Genotyping BC_1F_2 and phenotyping their BC_1F_3 populations map *BPH15* to interval between RM261 and S16; BC_2F_2 and BC_4F_2 plants were used to screen recombinants plant between RM261 and S16; BC_2F_3 of recombinants plants were used to find fixed recombinant plants; BC_2F_4 of fixed recombinants plants were used to phenotyping. Mentioned in the text were marked in red.
