# Supplementary material for: BAC and RNA sequencing reveal the brown planthopper resistance gene BPH15 in a recombination cold spot that mediates a unique defense mechanism
Source: BMC Genomics. 2014 Aug 11;15(1):674. doi: 10.1186/1471-2164-15-674 (PMC4148935; doi:10.1186/1471-2164-15-674)
Supplement: Supplementary file 6 — Additional file 6: K-means clustering analysis of DEGs based on log ratio of FPKM data. (PDF 401 KB) [file 12864_2014_6374_MOESM6_ESM.pdf]

A

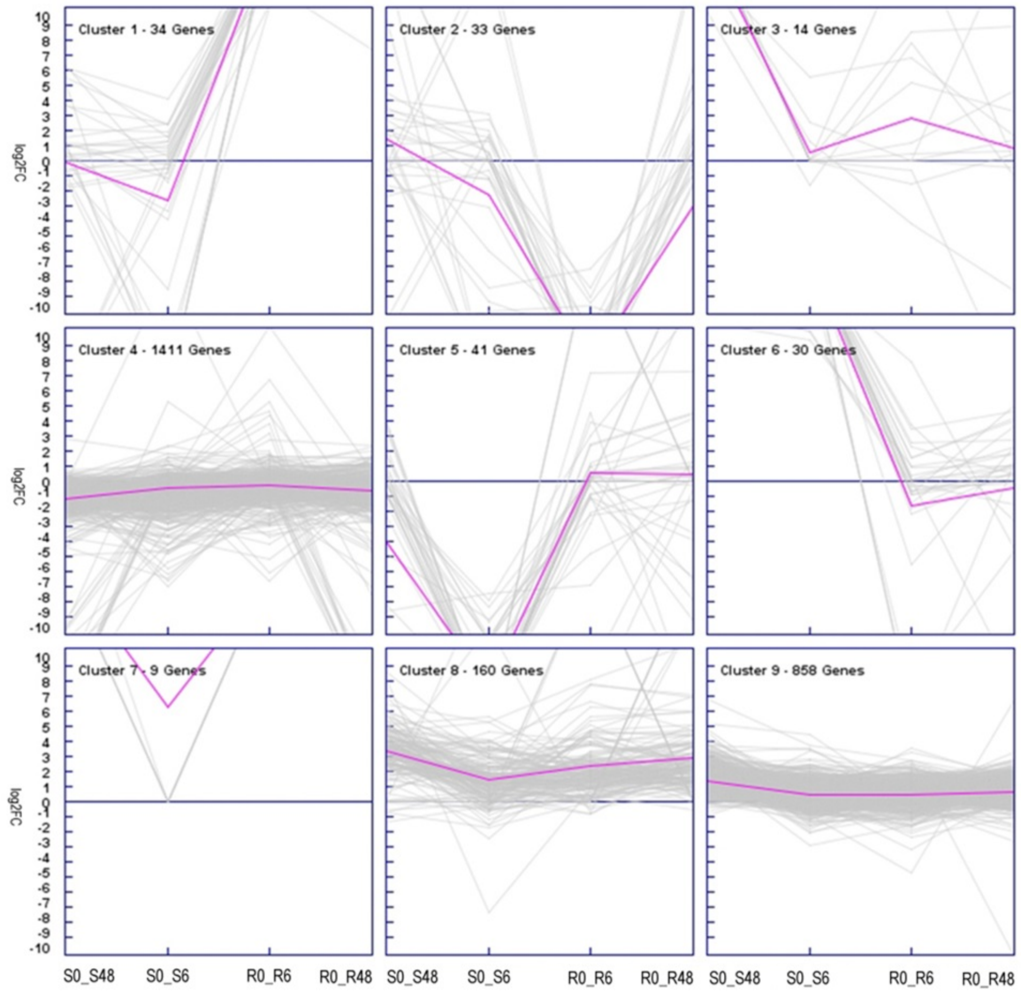

Cluster 1 - 34 Genes
Cluster 2 - 33 Genes
Cluster 3 - 14 Genes
Cluster 4 - 1411 Genes
Cluster 5 - 41 Genes
Cluster 6 - 30 Genes
Cluster 7 - 9 Genes
Cluster 8 - 160 Genes
Cluster 9 - 858 Genes
log2FC
S0_S48
S0_S6
R0_R6
R0_R48

B

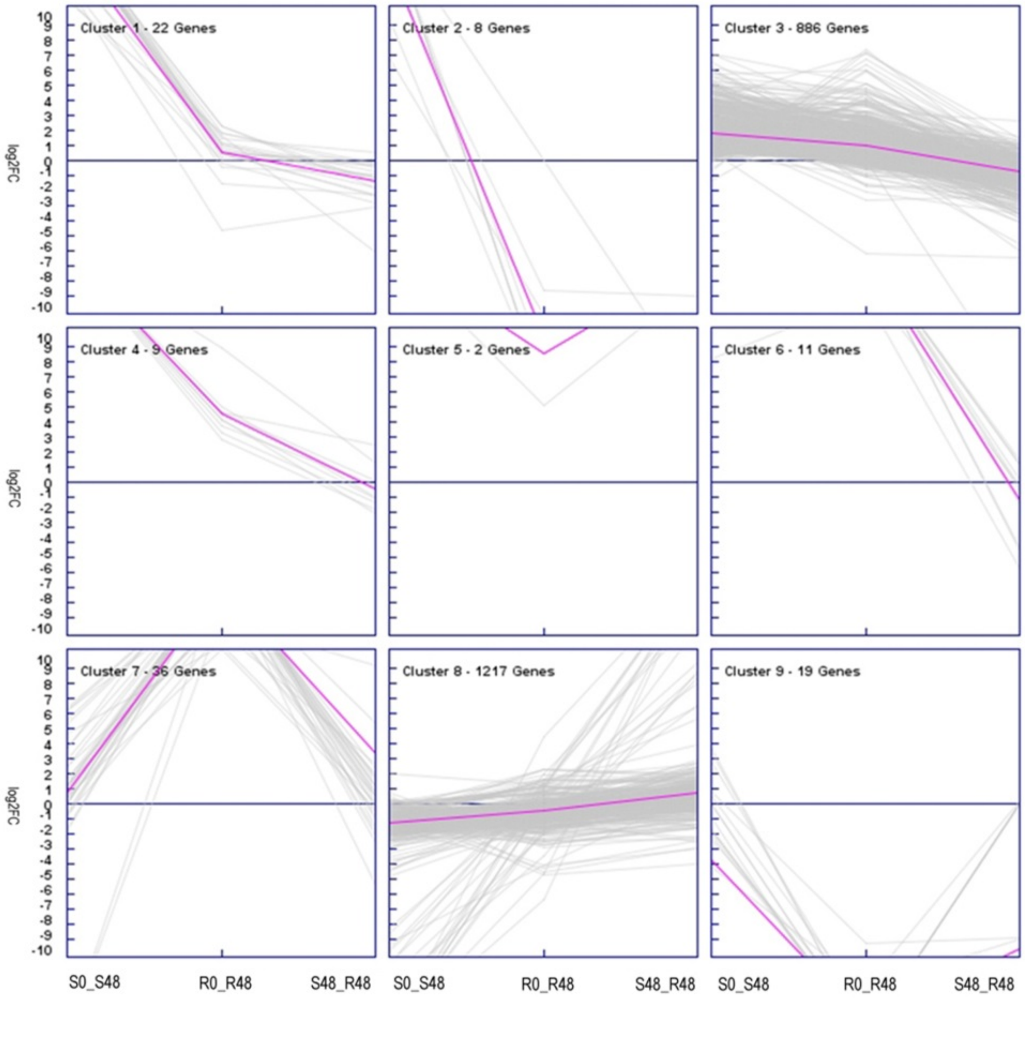

Cluster 1 - 22 Genes
Cluster 2 - 8 Genes
Cluster 3 - 886 Genes
Cluster 4 - 9 Genes
Cluster 5 - 2 Genes
Cluster 6 - 11 Genes
Cluster 7 - 36 Genes
Cluster 8 - 1217 Genes
Cluster 9 - 19 Genes
log2FC
S0_S48
R0_R48
S48_R48

Additional file 6 K-means clustering analysis of DEGs based on log ratio of FPKM data. A, DEGs of S and R. B, DEGs at 48 HAI. The pink lines indicate representative transcriptional regulators.
