# Supplementary material for: BAC and RNA sequencing reveal the brown planthopper resistance gene BPH15 in a recombination cold spot that mediates a unique defense mechanism
Source: BMC Genomics. 2014 Aug 11;15(1):674. doi: 10.1186/1471-2164-15-674 (PMC4148935; doi:10.1186/1471-2164-15-674)
Supplement: Supplementary file 9 — Additional file 9: RNA-seq concordance with real-time qPCR results. (PDF 99 KB) [file 12864_2014_6374_MOESM9_ESM.pdf]

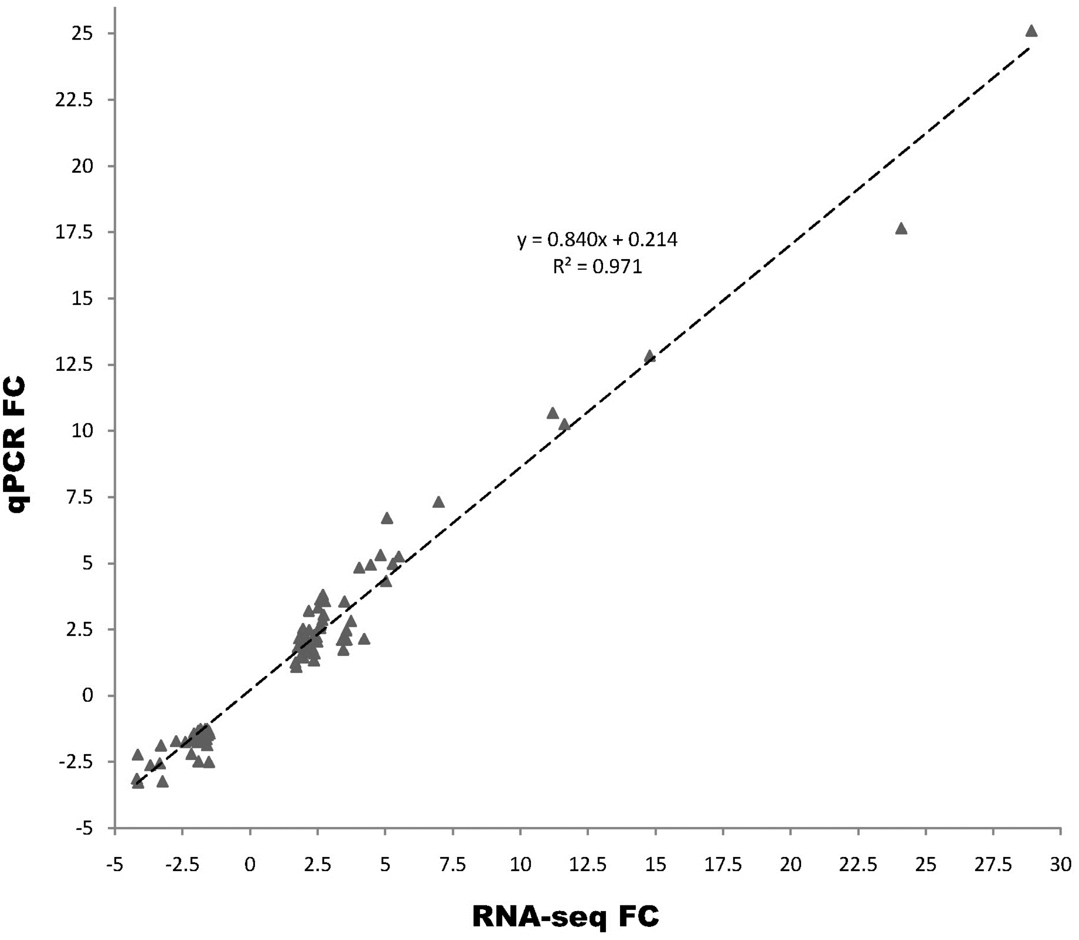

Additional file 9 RNA-seq concordance with real-time qPCR results. The graph displays the concordance between RNA-seq and qPCR fold-change values on a linear scale.
