# Supplementary material for: BAC and RNA sequencing reveal the brown planthopper resistance gene BPH15 in a recombination cold spot that mediates a unique defense mechanism
Source: BMC Genomics. 2014 Aug 11;15(1):674. doi: 10.1186/1471-2164-15-674 (PMC4148935; doi:10.1186/1471-2164-15-674)
Supplement: Supplementary file 10 — Additional file 10: Measurement of salicylic acid content using gas chromatography–mass spectrometry. (PDF 164 KB) [file 12864_2014_6374_MOESM10_ESM.pdf]

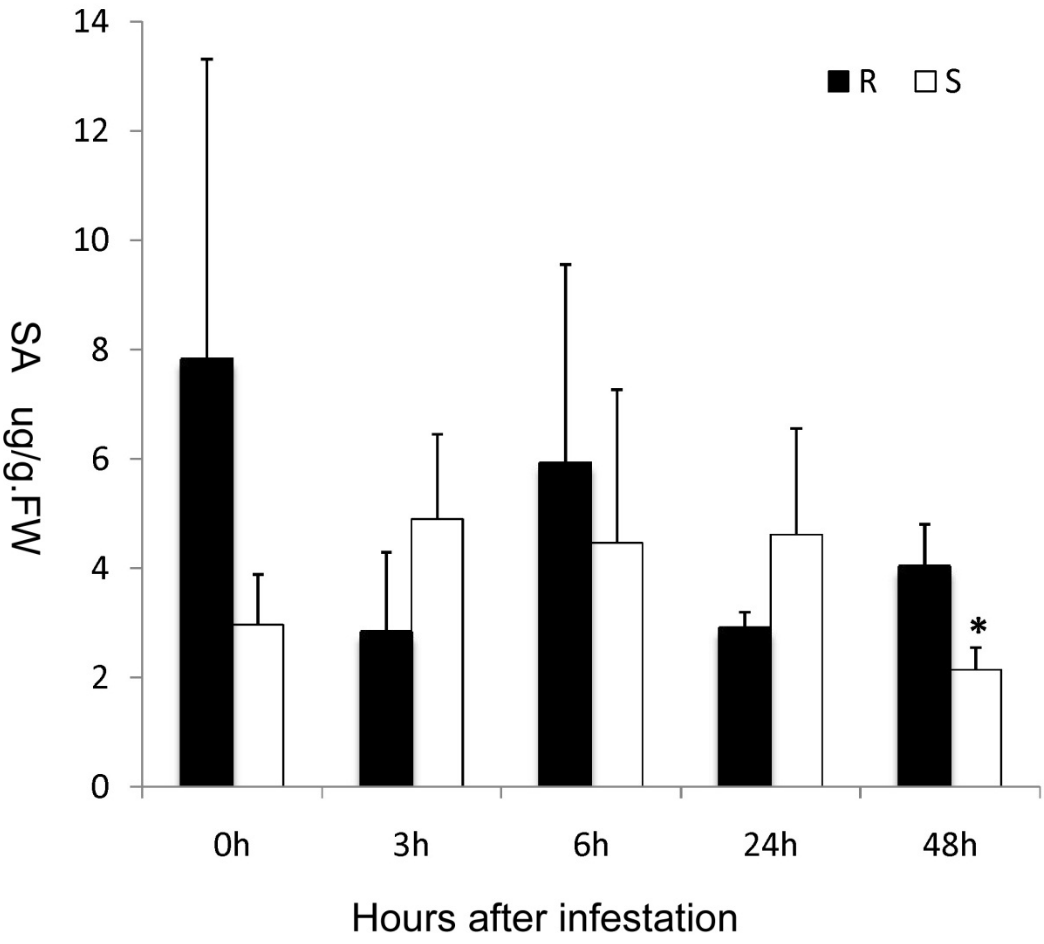

Additional file 10 Measurement of salicylic acid content using gas chromatography-mass spectrometry. Error bars represent the SD, *n* = 3.
