# Supplementary material for: BAC and RNA sequencing reveal the brown planthopper resistance gene BPH15 in a recombination cold spot that mediates a unique defense mechanism
Source: BMC Genomics. 2014 Aug 11;15(1):674. doi: 10.1186/1471-2164-15-674 (PMC4148935; doi:10.1186/1471-2164-15-674)
Supplement: Supplementary file 12 — Additional file 12: Graphical result of significant GO terms of S-all. (PDF 2 MB) [file 12864_2014_6374_MOESM12_ESM.pdf]

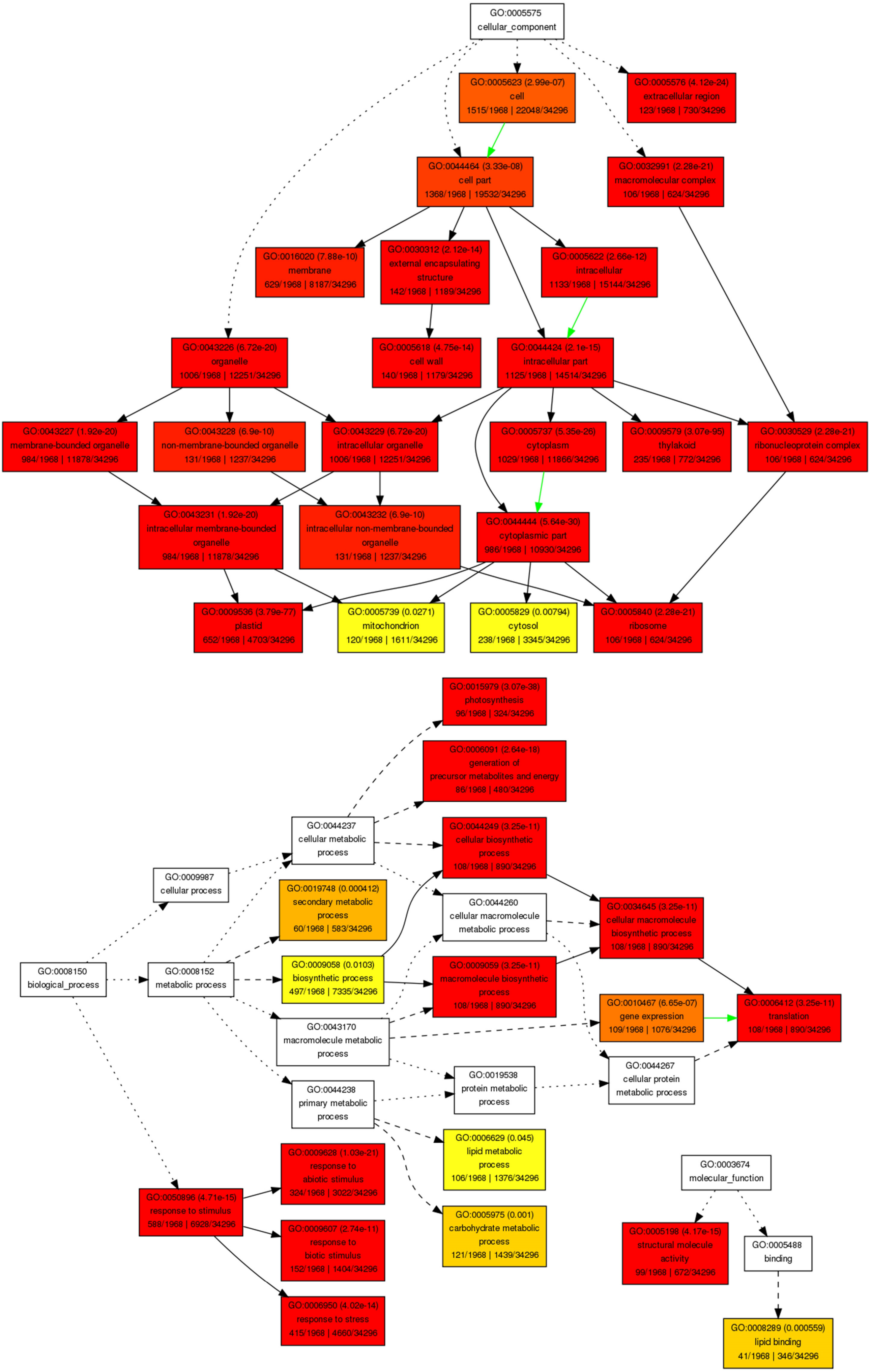

GO:0005575
cellular_component
GO:0005623 (2.99e-07)
cell
1515/1968 | 22048/34296
GO:0005576 (4.12e-24)
extracellular region
123/1968 | 730/34296
GO:0044464 (3.33e-08)
cell part
1368/1968 | 19532/34296
GO:0032991 (2.28e-21)
macromolecular complex
106/1968 | 624/34296
GO:0016020 (7.88e-10)
membrane
629/1968 | 8187/34296
GO:0030312 (2.12e-14)
external encapsulating
structure
142/1968 | 1189/34296
GO:0005622 (2.66e-12)
intracellular
1133/1968 | 15144/34296
GO:0043226 (6.72e-20)
organelle
1006/1968 | 12251/34296
GO:0005618 (4.75e-14)
cell wall
140/1968 | 1179/34296
GO:0044424 (2.1e-15)
intracellular part
1125/1968 | 14514/34296
GO:0043227 (1.92e-20)
membrane-bounded organelle
984/1968 | 11878/34296
GO:0043228 (6.9e-10)
non-membrane-bounded organelle
131/1968 | 1237/34296
GO:0043229 (6.72e-20)
intracellular organelle
1006/1968 | 12251/34296
GO:0005737 (5.35e-26)
cytoplasm
1029/1968 | 11866/34296
GO:0009579 (3.07e-95)
thylakoid
235/1968 | 772/34296
GO:0030529 (2.28e-21)
ribonucleoprotein complex
106/1968 | 624/34296
GO:0043231 (1.92e-20)
intracellular membrane-bounded
organelle
984/1968 | 11878/34296
GO:0043232 (6.9e-10)
intracellular non-membrane-bounded
organelle
131/1968 | 1237/34296
GO:0044444 (5.64e-30)
cytoplasmic part
986/1968 | 10930/34296
GO:0009536 (3.79e-77)
plastid
652/1968 | 4703/34296
GO:0005739 (0.0271)
mitochondrion
120/1968 | 1611/34296
GO:0005829 (0.00794)
cytosol
238/1968 | 3345/34296
GO:0005840 (2.28e-21)
ribosome
106/1968 | 624/34296
GO:0015979 (3.07e-38)
photosynthesis
96/1968 | 324/34296
GO:0006091 (2.64e-18)
generation of
precursor metabolites and energy
86/1968 | 480/34296
GO:0044237
cellular metabolic
process
GO:0044249 (3.25e-11)
cellular biosynthetic
process
108/1968 | 890/34296
GO:0009987
cellular process
GO:0019748 (0.000412)
secondary metabolic
process
60/1968 | 583/34296
GO:0044260
cellular macromolecule
metabolic process
GO:0034645 (3.25e-11)
cellular macromolecule
biosynthetic process
108/1968 | 890/34296
GO:0008150
biological_process
GO:0008152
metabolic process
GO:0009058 (0.0103)
biosynthetic process
497/1968 | 7335/34296
GO:0009059 (3.25e-11)
macromolecule biosynthetic
process
108/1968 | 890/34296
GO:0043170
macromolecule metabolic
process
GO:0010467 (6.65e-07)
gene expression
109/1968 | 1076/34296
GO:0006412 (3.25e-11)
translation
108/1968 | 890/34296
GO:0019538
protein metabolic
process
GO:0044267
cellular protein
metabolic process
GO:0044238
primary metabolic
process
GO:0006629 (0.045)
lipid metabolic
process
106/1968 | 1376/34296
GO:0009628 (1.03e-21)
response to
abiotic stimulus
324/1968 | 3022/34296
GO:0003674
molecular_function
GO:0050896 (4.71e-15)
response to stimulus
588/1968 | 6928/34296
GO:0005975 (0.001)
carbohydrate metabolic
process
121/1968 | 1439/34296
GO:0005198 (4.17e-15)
structural molecule
activity
99/1968 | 672/34296
GO:0005488
binding
GO:0009607 (2.74e-11)
response to
biotic stimulus
152/1968 | 1404/34296
GO:0006950 (4.02e-14)
response to stress
415/1968 | 4660/34296
GO:0008289 (0.000559)
lipid binding
41/1968 | 346/34296

Additional file 12 Graphical result of significant GO terms of S-all. The nodes in the image are classified into 10 levels, which are associated with corresponding specific colors. A smaller adjusted *P*-value indicates greater statistical significance, and the node's color is darker and redder. Information inside the box of the significant terms includes GO term, adjusted *P*-value, GO description, item number mapping the GO in the query list and background, and total number of query lists and background.
