# Supplementary figures and images for: BAC and RNA sequencing reveal the brown planthopper resistance gene BPH15 in a recombination cold spot that mediates a unique defense mechanism
Source: BMC Genomics. 2014 Aug 11;15(1):674. doi: 10.1186/1471-2164-15-674 (PMC4148935; doi:10.1186/1471-2164-15-674)

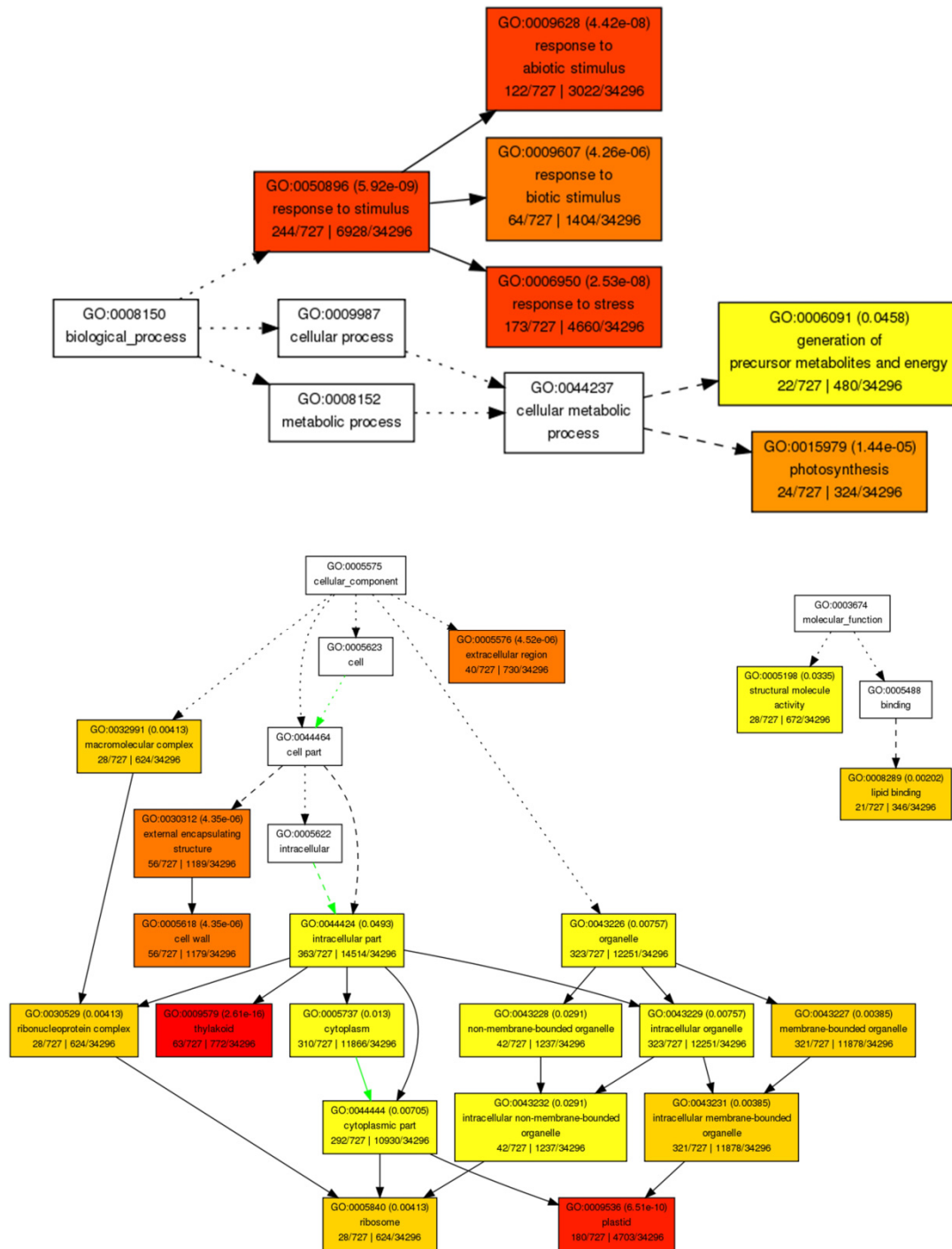

**Additional file 13** Graphical result of significant GO terms of R-all.

Supplement: Supplementary file 13 — Additional file 13: Graphical result of significant GO terms of R-all. (PDF 472 KB) [file 12864_2014_6374_MOESM13_ESM.pdf]

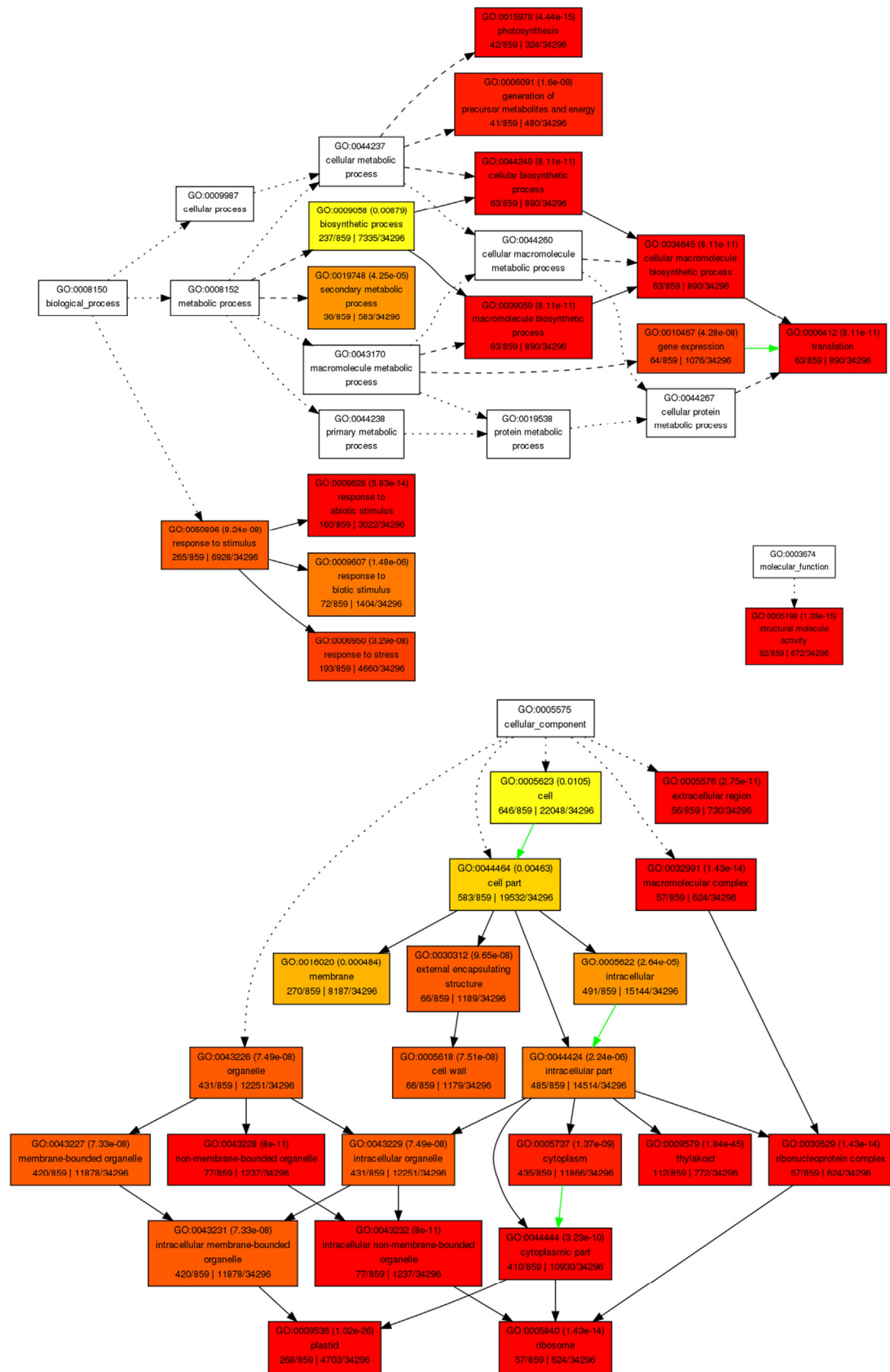

**Additional file 14** Graphical result of significant GO terms of S\_R-all.

Supplement: Supplementary file 14 — Additional file 14: Graphical result of significant GO terms of S_R-all. (PDF 774 KB) [file 12864_2014_6374_MOESM14_ESM.pdf]
